# Late presentation of Torsades de Pointes related to fluoxetine following a multiple drug overdose

**DOI:** 10.1186/s40560-018-0329-1

**Published:** 2018-09-10

**Authors:** Jan Albert Nicolaas Groot, Leonore ten Bokum, Hubertus Laurentius Antonius van den Oever

**Affiliations:** 1 Intensive Care Unit, Deventer Hospital, Nico Bolkesteinlaan 75, Deventer, 7416 SE The Netherlands; 2 Department of Clinical Pharmacy, Deventer Hospital, Nico Bolkesteinlaan 75, Deventer, 7416 SE The Netherlands

**Keywords:** Fluoxetine, Risperidone, Depression, Overdose, QTc prolongation, Torsades de Pointes

## Abstract

**Background:**

Fluoxetine is a selective serotonin reuptake inhibitor (SSRI) commonly used in the treatment of depression. While most intoxications with SSRI’s have favorable outcomes and do not require interventions other than strict observation of vital signs and heart rhythm, clinicians should be aware of the life-threatening complications that may occur.

**Case presentation:**

A 61-year-old woman presented to the emergency department after an intentional multiple drug overdose. Upon examination, she was somnolent with stable respiration and hemodynamics. Electrocardiography showed a prolonged QTc interval of 503 ms. The patient was admitted to the ICU for cardiopulmonary monitoring. During admission, the patient remained stable and showed improved neurologic function over time. After 22 h, a second ECG showed normalization of the QTc interval to 458 ms. However, 36 to 40 h after admission, our patient developed recurrent episodes of Torsades de Pointes (TdP) with loss of cardiac output, leading to cardiopulmonary resuscitation. Spontaneous circulation was restored after intravenous administration of magnesium sulphate. Retrospective serum analysis revealed fluoxetine concentrations of 2700 mcg/l.

**Conclusion:**

Most intoxications with selective serotonin reuptake inhibitors (SSRI) have favorable outcomes and do not require medical interventions other than strict cardiopulmonary observation. However, higher doses have been associated with QTc interval prolongation, TdP, serotonin syndrome, and death. This case illustrates that life-threatening complications may occur late in the course of hospital admission. Even though overdoses with SSRI’s generally result in few fatalities, clinicians should be aware of the life-threatening clinical manifestations that may occur. Despite being an imperfect predictor of imminent TdP, continuous monitoring of cardiac rhythm is strongly recommended when either cardiac or non-cardiac symptoms are present.

## Background

In 1966, French cardiologist François Dessertenne described a distinct polymorphic ventricular tachycardia in patients with QTc prolongation that exhibits specific characteristics on the electrocardiogram [1]. Torsades de Pointes (“twisting of the points”) is characterized by rapid, irregular QRS complexes “twisting” around the isoelectric baseline. Depending on the cause, TdP may either spontaneously revert back into sinus rhythm or degenerate into other life-threatening dysrhythmias. When nonsustained, it frequently reoccurs if the underlying cause is not treated adequately. Moreover, TdP may evolve into sustained ventricular fibrillation leading to hemodynamic compromise and eventually death. In this report, we describe an unsuspected case of TdP in a patient recovering from a multiple drug overdose.

## Case presentation

**Fig. 1:**
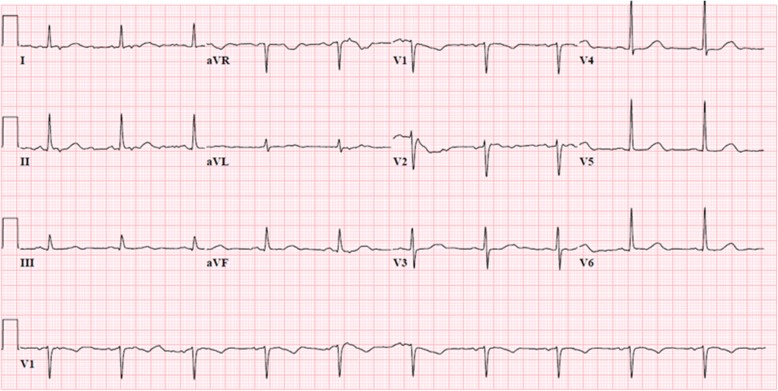
Initial electrocardiogram showing a sinus rhythm with a prolonged QTc interval of 503 ms. The tangent method was used in order to define the end of the T-wave

A 61-year-old woman with a past medical history of type II diabetes, breast cancer, and major depression presented to the emergency department after an intentional overdose with fluoxetine (139 tablets of 20 mg), risperidone (6 tablets of 1 mg), bromazepam (90 tablets of 3 mg), zolpidem (40 tablets of 10 mg), naproxen (20 tablets of 500 mg), and clemastine (5 tablets of 1 mg). Quantities were determined by counting the remaining pills in the blister packaging. Upon arrival, the patient was somnolent but able to open her eyes on request (E3M6V5). Vital signs showed a blood pressure of 146/57 mmHg, a regular heart rate of 55/min with strong peripheral pulsations, a respiratory rate of 16/min, oxygen saturation levels varying between 95 and 100% at room air, and a body temperature of 36.2 °C. Her husband suggested that the pills must have been ingested 3 to 8 h prior to hospital admission. Electrocardiography (ECG) showed a sinus rhythm of 61 beats per minute with a prolonged corrected QT interval (QTc) of 503 ms as shown in Fig. 1. During admission, all ECGs were performed using a GE MAC 5500 HD electrocardiograph. The tangent method was used in order to define the end of the T-wave in the lead with the longest QT interval. All measured QTc intervals were corrected for cardiac frequency using Bazett’s formula [2].

Initial laboratory findings showed a microcytic anemia (Hb 5.4 mmol/l, MCV 77 fl) with low serum ferritin (6 μg/l), along with a mild leukocytosis of 11.2/nl. Serum electrolytes, as well as liver and kidney function, were normal (Na^+^ 135 mmol/l, K^+^ 4.3 mmol/l, creatinine 89 μmol/l, corrected calcium 2.37 mmol/l, PO_4_^3−^ 1.41 mmol/l, magnesium 0.83 mmol/l). Arterial blood gas analysis showed a base excess of − 4.9 mmol/l (pH 7.37, pCO_2_ 4.6 kPa, pO_2_ 12.1 kPa, HCO_3_^−^ 20 mmol/l). A chest X-ray displayed no aspiration pneumonia nor other cardiopulmonary anomalies. The patient was admitted to the ICU for observation, where she received intravenous fluids along with 40 mg of pantoprazole for the prevention of peptic ulcer disease due to the substantial ingestion of naproxen. This was administered only once due to the risk of further QTc interval prolongation. Initially, our patient remained hemodynamically stable and showed improved neurological function. Twenty-two hours after hospital admission, a second ECG showed normalization of the QTc interval to 458 ms, suggesting peak serum levels of the ingested drugs had passed. After psychiatric evaluation had taken place, our patient had fallen to the floor in search of the restroom. On examination, both her muscle strength and coordination were slightly disturbed. Due to the overall severity of the intoxication and the persistence of neurological symptoms, it was decided to observe the patient for one more night at the ICU. Flumazenil was not administered since the patient was fully conscious and already in a monitored environment and due to the risk of adverse effects, especially with chronic benzodiazepine use [3].

**Fig. 2:**
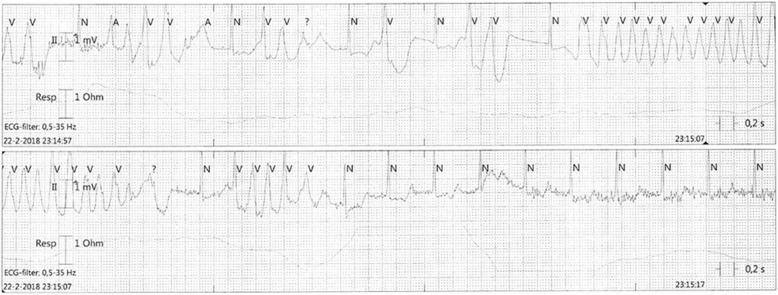
Telemetry records showing Torsades de Pointes

**Fig. 3:**
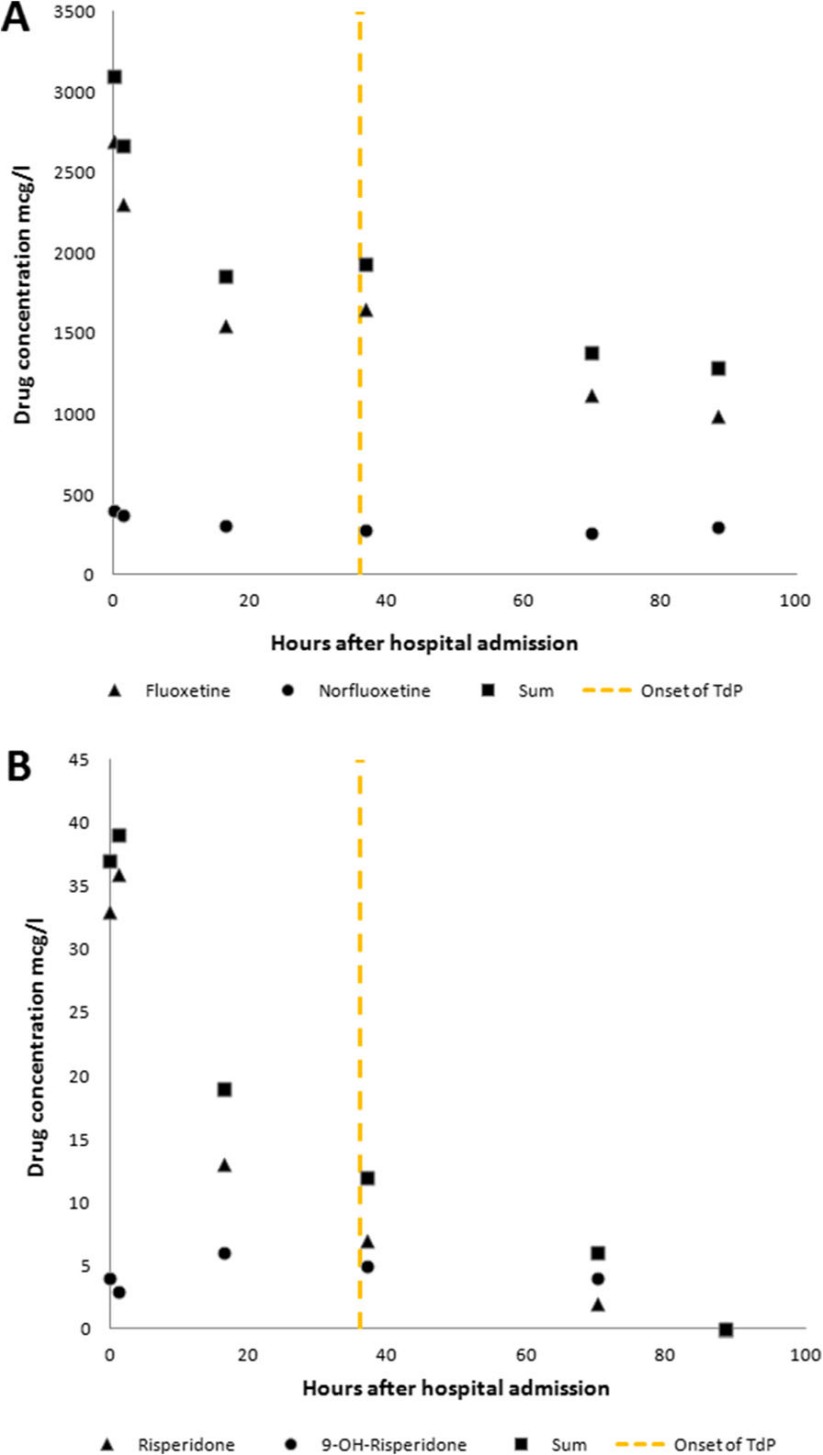
**a** Showing serum drug serum levels of fluoxetine(black triangles), norfluoxetine (black circles), and the combined serum concentrations of both fluoxetine and norfluoxetine (black squares). **b** Showing drug serum levels of risperidone (black triangles), 9-OH-risperidone (black circles), and the combined concentrations of both risperidone and 9-OH-risperidone (black squares). The yellow striped line indicates the onset of TdP

**Fig. 4:**
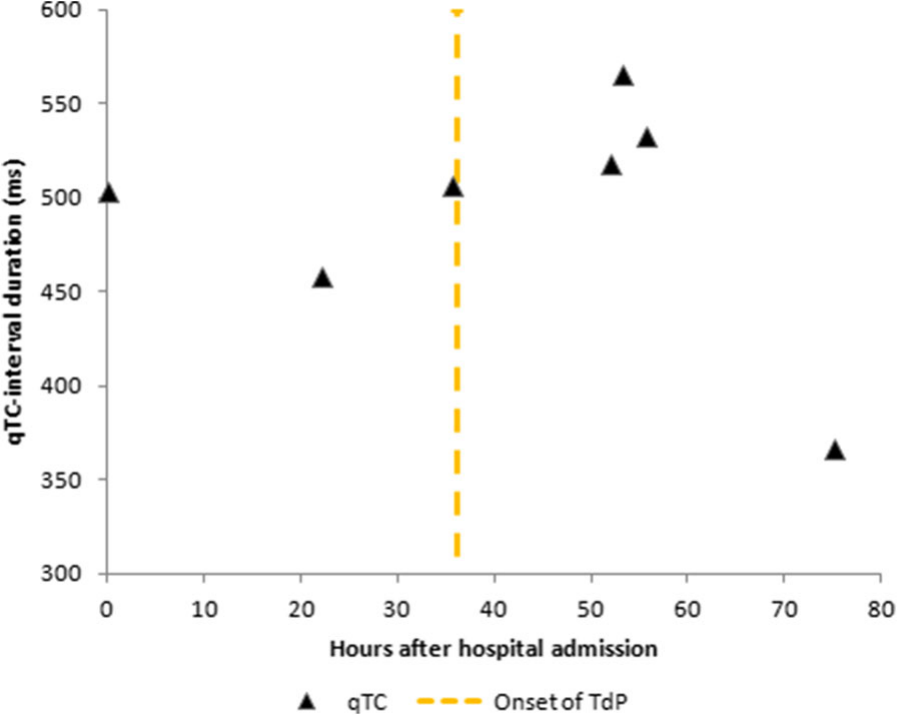
Showing the course of QTc interval duration during hospital admission

That night, 36 h after admission, our patient developed recurrent short episodes of Torsades de Pointes (TdP) with intermittent loss of cardiac output, as shown in Fig. 2. Two grams of magnesium sulphate was administered intravenously, after which sinus rhythm was restored. Four hours later, she had complete loss of circulation, after which cardiopulmonary resuscitation was initiated according to protocol. A 150-J biphasic shock was delivered using an automated external defibrillator (Zoll R-Series ALS). ECG findings consistent with TdP were again observed. Spontaneous circulation was restored after intravenous administration of magnesium sulphate. Our patient was awake and immediately able to maintain a patent airway following the incident. Serum levels of fluoxetine and risperidone and their metabolites were measured in retrospect, as shown in Table 1 and Fig. 3a, b. Laboratory findings at the time of the incident showed a mild hypocalcaemia of 2.10 mmol/l with a hypermagnesaemia of 1.72 mmol/l/, most likely caused due to the prior administration of magnesium sulphate. No other electrolyte abnormalities were identified (Na^+^ 137 mmol/l, K^+^ 3.8 mmol/l, PO_4_^3−^ 1.24 mmol/l). Subsequent ECGs showed progressive prolongation of the QTc interval up to 565 ms, as shown in Fig. 4. The patient remained hemodynamically stable for the remainder of the night on continuous infusion of magnesium sulphate (1 g/h). The following day, echocardiography was performed in order to exclude structural cardiac defects, which showed normal heart dimensions as well as a normal left and right ventricular function.

**Table 1:** Serum drug concentration levels of (nor)fluoxetoine and (9-OH)risperidone during hospital admission

Hours after hospital admission	Fluoxetine (ref 100–450 mcg/l)	Norfluoxetine (ref 50–350 mcg/l)	Sum (ref 15*–*500 mcg/l)	Risperidone (ref 4*–*30 mcg/l)	9-OH-risperidone (ref 12*–*60 mcg/l)	Sum (ref 20–60 mcg/l)
*T* = 0	2700	397	3097	33	4	37
*T* + 1.33	2300	370	2670	36	3	39
*T* + 16.3	1550	310	1860	13	6	19
*T* + 36.9	1650	281	1931	7	5	12
*T* + 70.0	1120	263	1383	2	4	6
*T* + 88.5	990	300	1290	NA	NA	NA

## Discussion

QT interval prolongation can either be congenital or, more commonly, drug-induced [4]. Apart from genetic predispositions, several risk factors have shown to increase a patient’s likelihood of developing TdP. Examples include electrolyte abnormalities, hypothermia, bradycardia, (congenital) heart disease, and the female sex [5]. When QT prolongation is present, immediate withdrawal of agents that may contribute to QT interval prolongation as well as prompt correction of underlying electrolyte disturbances is indicated. TdP leading to hemodynamic instability is primarily treated by electrical cardioversion. However, in stable patients with recurrent self-terminating episodes of TdP, administration of intravenous magnesium sulphate is the therapy of choice [6, 7].

Congenital long QT syndrome (LQTS) is a condition which affects cardiac repolarization due to specific genetic alterations, leading to an increased risk of developing arrhythmias leading to syncope and possibly cardiac arrest [8]. In contrast to congenital long QT syndrome, drug-induced QT prolongation involves blocking of the delayed rectifier potassium current (I_Kr_) by disrupting hERG potassium channels, thereby extending the repolarization phase of the myocardial cells resulting in QT prolongation [4].

Drug-induced QTc prolongation along with the occurrence of TdP is associated with a wide range of drugs including class III repolarization-delaying antiarrhythmics, antipsychotics, and antimicrobial agents [4]. Previous to admission, our patient had ingested substantial amounts of fluoxetine and risperidone as well as zolpidem and clemastine. Although reports exist that suggest an association between zolpidem, clemastine, and QT prolongation, those reports are limited in number. Both zolpidem and clemastine do not appear on the 2018 AZCERT list of accepted QT prolongators [9–11]. Therefore, in this report, we focused on the QT-prolonging properties of fluoxetine and risperidone.

Fluoxetine is a selective serotonin reuptake inhibitor used in the treatment of major depression. It reaches its *T*_max_ 6–8 h after administration and is metabolized mainly by CYP2D6 into its pharmacologically active metabolite norfluoxetine [12]. Both fluoxetine and norfluoxetine have shown to be potent inhibitors of CYP2D6 [13]. Fluoxetine has a half-life of 4–6 days, while its metabolite norfluoxetine has a half-life varying from 4 to 16 days. Risperidone is an atypical antipsychotic with strong anti-dopaminergic (D2) and anti-serotonergic (5Ht2) activity [14]. *T*_max_ is reached 1–2 h after administration and is also metabolized by CYP2D6 into the active 9-hydroxyrisperidone. When compared to fluoxetine, both risperidone and 9-OH-risperidone have relatively short half-lives of 3 and 24 h respectively. Theoretically, (nor)fluoxetine-mediated CYP2D6 inhibition can result in higher than expected risperidone serum levels, causing adverse effects.

We hypothesized that the sudden clinical deterioration might have been caused by the accumulation of either fluoxetine, risperidone, or their respective metabolites, resulting in an increased susceptibly for additional risk factors as stated above. In order to test this hypothesis, serum levels of fluoxetine, norfluoxetine, risperidone, and 9-OH-risperidone were determined retrospectively using previously drawn blood samples (Fig. 3a, b). Furthermore, QTc interval durations were obtained from all electrocardiographs made during hospital admission and plotted as displayed in Fig. 4. Plasma serum levels of fluoxetine reached high concentrations of up to 2700 mcg/l, which declined gradually over time. Neither risperidone nor 9-OH-risperidone reached toxic serum concentrations. Both fluoxetine and risperidone serum levels peaked during the first hours of hospital admission. However, at that point, our patient showed no signs of cardiac impairment besides a prolonged QTc interval of 503 ms. On subsequent days, increasing QTc intervals up until 565 ms were measured 54 h after admission. During this time, we found no evidence for an accumulation of either (nor)fluoxetine or (9-OH)risperidone, suggesting factors other than fluoxetine and risperidone drug concentrations caused the onset of TdP in our patient.

Despite being a key element in the development of TdP, QTc prolongation alone is considered insufficient to cause TdP [15]. Additional risk factors such as electrolyte abnormalities, hypothermia, (extreme) bradycardia, heart disease, and the female sex are believed to be contributing factors in the development of TdP. Unfortunately, except for the female sex and a mild hypocalcaemia, no additional risk factors were identified in our patient. Although hypocalcemia has been associated with QTc prolongation, the number of cases is limited and characterized by significantly lower serum calcium concentrations, making it an unlikely cause of TdP in our patient [16, 17]. Proton pump inhibitors (PPIs) have also shown to result in QT prolongation secondary due to the development of hypomagnesaemia [18]. However, serum magnesium concentrations in our patient were repeatedly measured to be within normal range. As a result, we considered the prior administration of pantoprazole an improbable cause of recurrent TdP. Sixteen hours after developing TdP, electrocardiography showed further QTC prolongation from 506 to 518 ms. Two hours later, the QTc interval reached its peak of 565 ms. Therefore, the prolonged QTc interval could not have been secondary to the ventricular arrythmia that had occurred 18 h earlier. It is possible that just before the onset of TdP, the QTc interval was prolonged even more than 506 ms. Unfortunately, during that period, no additional ECGs were performed. While possible, it is unlikely that our patient is genetically predisposed to LQTS, since ECGs from previous admissions showed normal QTc intervals. Moreover, family history was negative for LQTS and sudden cardiac arrest. It therefore seems evident that our patient developed QTc interval prolongation due to a substantial overdose of fluoxetine. Unfortunately, we were unable to identify any contributing risk factor(s) besides a mild hypocalcaemia and female sex. Moreover, it still remains unclear why the initial decline in QTc interval duration and the improvement of clinical symptoms were followed by QTc prolongation and the development of cardiac arrhythmias. This further highlights why extensive heart rhythm control and intermittent electrocardiography as well as monitoring of serum electrolytes should be considered mandatory in caring for a patient suffering from an overdose resulting in QTc prolongation.

## Conclusion

We described a case of intentional drug overdose with fluoxetine resulting in QT prolongation and the development of TdP. While most overdoses with selective serotonin reuptake inhibitors have favorable outcomes and do not require medical interventions other than cardiopulmonary observation, clinicians should be aware of the potentially life-threatening complications that may occur. High-dose intoxications have been associated with QT prolongation, TdP, and eventually, death. This case illustrates that both measurement of serum drug concentrations and monitoring of the QT interval are imperfect indicators of imminent cardiac dysrhythmias. Increasing evidence shows that additional risk factors such as electrolyte disturbances are required to trigger TdP. Even though serum drug monitoring is considered unessential in caring for patients suffering from multiple drug overdoses, it may provide insight in estimating toxicity levels when pharmacokinetic and pharmacodynamic drug interactions are suspected. Extensive heart rhythm control, intermittent electrocardiography, and monitoring serum electrolytes are strongly recommended as long as any cardiac or non-cardiac symptoms are still present.

## Abbreviations

CYP2D6: Cytochrome P450 family 2 subfamily D member 6
ECG: Electrocardiogram
Hb: Hemoglobin
ICU: Intensive care unit
LQTS: Long QT syndrome
MCV: Mean corpuscular volume
SSRI: Selective serotonin reuptake inhibitor
TdP: Torsades de Pointes
